# Consensus on Changing Trends, Attitudes, and Concepts of Asian Beauty

**DOI:** 10.1007/s00266-015-0562-0

**Published:** 2015-09-25

**Authors:** Steven Liew, Woffles T. L. Wu, Henry H. Chan, Wilson W. S. Ho, Hee-Jin Kim, Greg J. Goodman, Peter H. L. Peng, John D. Rogers

**Affiliations:** Shape Clinic, Sydney, Australia; Woffles Wu Aesthetic Surgery and Laser Centre, Camden Medical Centre, 1 Orchard Boulevard, Suite #09-02, Singapore, 249615 Singapore; Department of Medicine, University of Hong Kong, Pok Fu Lam, Hong Kong; The Specialists: Lasers, Aesthetic and Plastic Surgery Central, Pok Fu Lam, Hong Kong; Division of Anatomy & Developmental Biology, Department of Oral Biology, Yonsei University College of Dentistry, Seoul, Korea; Monash University, Clayton, Victoria, and Skin & Cancer Foundation, Carlton, VIC Australia; P-Skin Professional Clinic, Kaohsiung, Taiwan; Regional Medical Affairs, Allergan Asia Pacific, Singapore, Singapore

**Keywords:** Asian facial esthetics, Asian facial features, Asian facial aging, Asian facial anatomy, Consensus opinion, Facial injectables

## Abstract

**Background:**

Asians increasingly seek non-surgical facial esthetic treatments, especially at younger ages. Published recommendations and clinical evidence mostly reference Western populations, but Asians differ from them in terms of attitudes to beauty, structural facial anatomy, and signs and rates of aging. A thorough knowledge of the key esthetic concerns and requirements for the Asian face is required to strategize appropriate facial esthetic treatments with botulinum toxin and hyaluronic acid (HA) fillers.

**Methods:**

The Asian Facial Aesthetics Expert Consensus Group met to develop consensus statements on concepts of facial beauty, key esthetic concerns, facial anatomy, and aging in Southeastern and Eastern Asians, as a prelude to developing consensus opinions on the cosmetic facial use of botulinum toxin and HA fillers in these populations.

**Results:**

Beautiful and esthetically attractive people of all races share similarities in appearance while retaining distinct ethnic features. Asians between the third and sixth decades age well compared with age-matched Caucasians. Younger Asians’ increasing requests for injectable treatments to improve facial shape and three-dimensionality often reflect a desire to correct underlying facial structural deficiencies or weaknesses that detract from ideals of facial beauty.

**Conclusions:**

Facial esthetic treatments in Asians are not aimed at Westernization, but rather the optimization of intrinsic Asian ethnic features, or correction of specific underlying structural features that are perceived as deficiencies. Thus, overall facial attractiveness is enhanced while retaining esthetic characteristics of Asian ethnicity. Because Asian patients age differently than Western patients, different management and treatment planning strategies are utilized.

**Level of Evidence V:**

This journal requires that authors assign a level of evidence to each article. For a full description of these Evidence-Based Medicine ratings, please refer to Table of Contents or the online Instructions to Authors www.springer.com/00266.

## Introduction

In Asia, the past decade has witnessed a tremendous increase in the number of patients who request and receive facial injectable treatments, non-ablative skin resurfacing and other non-surgical procedures, compared with the number of patients undergoing esthetic facial surgery. This is probably because the public has a much greater awareness of the treatment options available to them. Reasons for this include technological advances and improved results achievable with injectable treatments such as botulinum toxin and hyaluronic acid (HA) fillers, the increasing social acceptability of enhancing one’s appearance, increasing affordability and accessibility of injectable treatments, and the rise of the middle class in Asia.

Most Asian esthetic patients, whether young or old, prefer to avoid surgery wherever possible, and they seek natural-looking results. Therefore, Asian physicians have had to respond to their patients’ expectations, study, and then innovate procedures and management strategies to address the Asian esthetic, including facial shape, structure and proportion, and impact of the aging process on Asian faces.

To date, most studies and published recommendations on the use of facial injectable treatments (especially their use in combination) reference Western populations [1–7]. However, ethnic Asians differ from them in both facial appearance and baseline structural facial anatomy [8–12]. The signs and rate of onset of facial aging are also different in Asians [13–15]. Existing published recommendations cannot be applied directly to Asians. Furthermore, relatively few published papers cited in PubMed describe the use of botulinum toxin and HA fillers in Asians, and only one paper describes their combined use in the Asian face [16]. Unfortunately, data from clinical trials are often not relevant to real-world practice because typically only one standardized treatment intervention is studied in one facial area; however, esthetic treatment is usually multimodal and individualized. Therefore, there is a need for expert guidance on facial esthetic treatment of Asians.

To this end, the Asian Facial Aesthetics Expert Consensus Group, which comprised an anatomist, plastic surgeons, and dermatologists from 11 Asia–Pacific countries, met to discuss current practices regarding the use of non-invasive esthetic treatments in Asians. As a prelude to developing consensus opinions on the use of botulinum toxin and HA fillers in Asians, the group discussed concepts of facial beauty and attractiveness, as well as key esthetic concerns, facial anatomy, and aging in Southeastern and Eastern Asians. The Expert Group’s goal was to identify esthetic treatments and outcomes that Asian patients most commonly require, and to develop consensus opinions on how these can best be provided.

Proceedings of this meeting are intended to offer guidance to physicians who provide surgical and non-invasive facial esthetic treatment to Asian patients, in the absence of published clinical evidence. In this, the first of two papers, attitudes to facial beauty in Asia are described. Given that a thorough knowledge of the patient’s facial anatomy and aging process is required to inform facial esthetic treatment, those factors specific to Asian population groups are also discussed. Asians are defined here as the diverse groups of ethnicities from East Asia (e.g., China, Korea, Japan, Hong Kong, Taiwan) and Southeast Asia (e.g., Thailand, Singapore, Indonesia, Philippines); those from the Indian subcontinent are not included.

## Methods of Consensus Development

To determine the key trends in the type of Asian patients who present for facial esthetic treatment, the patients’ key facial esthetic concerns, and the most commonly used facial esthetic treatments for each age group, 25 members of the Expert Group completed a pre-meeting online survey developed by Dr. Steven Liew. Twenty-one Expert Group members then attended a consensus meeting in Seoul, Korea held on June 4 to June 5, 2014.

The members of this Expert Group have a mean 17 years of specialized experience in the field of facial esthetics (range 7–30 years) and treat Asian patients from China, Hong Kong, India, Indonesia, Japan, Korea, the Philippines, Singapore, Taiwan, Thailand, and Australia.

The process used to develop the consensus statements presented here was based on agreed statements created following discussions around survey outcomes, peer-reviewed literature, and clinical experience. Final versions of the statements were approved by all authors after being suggested and debated by the experts during the meeting, and modified, if necessary, while drafting the manuscript.

This article does not contain any studies with human participants or animals performed by any of the authors.

## Results: Consensus Outcomes

The points presented here are a summary of the outcomes of discussions that took place at the Expert Consensus Group meeting and thus reflect the consensus expert opinions of all participants.

### Concepts of Facial Beauty

Attractive and beautiful people of all races have distinct ethnic features, which reflect harmony, symmetry, and balance. However, when comparing the most attractive and beautiful people with their counterparts in other parts of the world, they share remarkable similarity with respect to facial shape [17, 18].

Facial shape is the essential key to facial beauty, with an oval face considered attractive (and youthful) by people of all racial backgrounds [17, 19, 20]. An oval face in this context refers to a smooth egg-shaped curve outlining the perimeter of the face, with a smooth transition from the forehead through the temples, around the outside of the cheeks, preauricular region, angle of the jaw, and jawline through to the chin, without indentations or projections in the line. A well-projected nose and chin is also considered attractive. Asians of all ethnicities and ages highly prize clear, unblemished, fair, and youthful skin.

Beauty is universal. Every ethnic group has its esthetically strong and weak points, but on the whole, the most beautiful and attractive people of each and all races tend to look similar in terms of face shape, and harmonious delicacy of features, balance, and symmetry. As faces become less attractive, they display more distinct ethnic features. Caucasian faces generally have more pronounced three-dimensionality with larger, more deeply set eyes, greater anterior projection of the brow, nose, maxilla, and chin. Caucasians also tend to have narrower faces and greater vertical height. Asians tend to have a wider face with shorter vertical height, which is flat or concave in the medial maxilla and has a lack of brow, nasal, and chin projection. On the other hand, they possess greater infraorbital volume, fuller lips, and superior skin qualities compared to Caucasians, which enables them to resist environmental insults and delay physiological and anatomical signs of aging.

Mindful of this concept of universal beauty, regardless of race or ethnicity, physicians in Asia seek to enhance “deficient” features and improve esthetic balance. In Asians, attractiveness is achieved by aiming to create an oval facial shape, by narrowing the lower face and increasing vertical height of the face. The anterior projection of the brow, medial cheek, nose, and chin is increased to improve the three-dimensionality of the face, and the appearance of the eyes is enlarged [20–22].

### Globalization and Changing Attitudes to Beauty

**Fig. 1:**
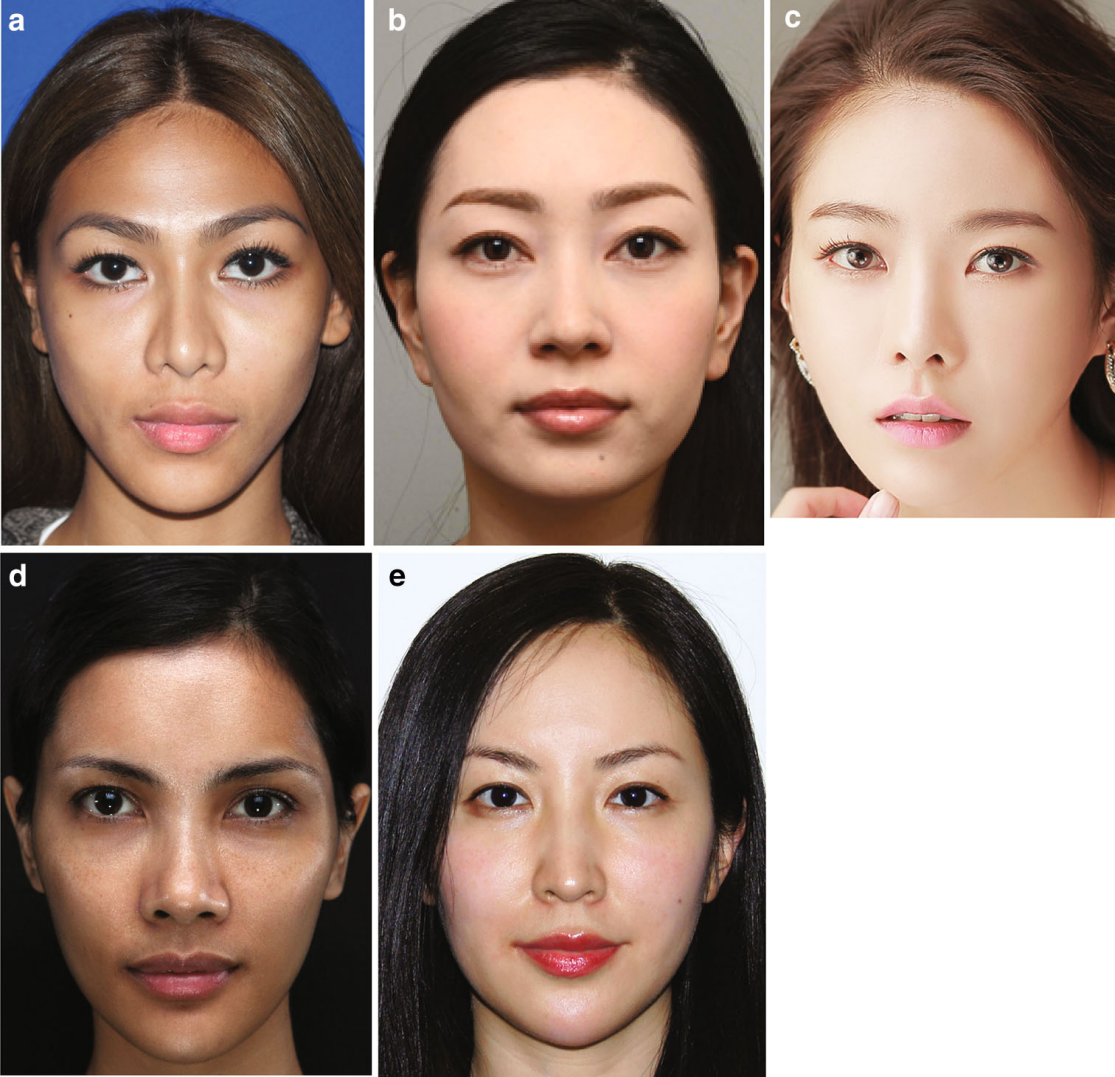
Examples of ethnic Asian beauty in women from **a** the Philippines (photo courtesy of Dr. Herve Raspaldo); **b** Japan (photo courtesy of Dr. Akiko Imizumi); **c** Korea (photo courtesy of Dr. Kyle Seo); **d** Indonesia (photo courtesy of Dr. Steven Liew); and **e** China (photo courtesy of Dr. Steven Liew)

Globalization has enhanced our ability to recognize, study, and create beauty in all ethnic groups (Fig. 1). In Asians, it is now common to improve anterior projection and three-dimensionality (double eyelid, nose/cheek/forehead augmentation), increase vertical height (chin augmentation), and reduce lower facial width (masseter reduction). In the past, these practices may have been perceived as an attempt to “Westernize” the Asian face. However, this is now understood to be the optimization of facial esthetic appearance within the individual’s own ethnicity.

Beauty is influenced by customs, traditions, and trends, and the current ideals for the female Asian face include a smooth, convex forehead, large eyes, a petite nose with a raised bridge, full but not prominent lips that are proportionally balanced, and an oval, egg-shaped face with a neat “v-shaped” jawline.

### Consensus Statements on Concepts of, and Attitudes to, Beauty

- Beautiful people of all races show similarity in facial characteristics while retaining distinct ethnic features.
- Asians are not a homogeneous group but rather comprise many varied ethnic origins, with each group having its own unique facial characteristics.
- Treatment to achieve esthetic changes in Asians should not be viewed as an attempt at Westernization, but rather the optimization of Asian ethnic features, in the same way that Westerners who receive lip enhancement, lateral malar enhancement, or skin tanning are not trying to “Easternize” their appearance as they attempt to make up for their intrinsic ethnicity-associated structural weaknesses.

### Evolving Treatment Trends in Asia

The desire for facial esthetic improvement has always existed, but uptake was previously limited due to the expense of plastic surgery and the limited number of skilled practitioners.

The results of the Expert Group’s pre-meeting survey showed that most Asian facial esthetics patients are female, but the proportion of males seeking treatment has increased during the last decade, from 12 % in 2005–2009 to 19 % in 2010–2014. The proportion of younger Asian patients (i.e., aged 18–40 years) who present with esthetic concerns has increased slightly in the past 5 years: from 44 % in 2005–2009 to 48 % in 2010–2014. This may be the result of an increased sense of self identity and pride, and younger patients having more economic power, aspiration, and social independence. Other reasons for this increase include (i) understanding that early use of esthetic treatment may prevent or reduce progress of aging; (ii) increasing awareness of esthetic procedures and treatments received by their peers and “public” figures in social media; (iii) increasing accessibility and affordability of products and treatments; (iv) increasing numbers of trained esthetic physicians; and (v) the safety of injectable products that has emerged over the past 5–10 years.

### Key Esthetic Concerns in Asia

The Expert Group’s pre-meeting survey responses regarding the top three structural esthetic concerns among Asians of different age groups is shown in Table 1—rated according to *physicians’ opinions* of what patients needed to have treated and according to what *physicians thought were the patients’ opinions* regarding their most pressing concerns. There was generally agreement between the two sets of opinions.

**Table 1:** Most common esthetic concerns among Asian patients – physicians’ opinions and physicians’ opinions of patients’ priorities

Priority	Opinion	Patient age group			
		18–30 years	31–40 years	41–55 years	>55 years
1	Physician	Nasal shape	Tear trough	Tear trough/Malar volume loss	Malar volume loss/jowls
	Patient	Nasal shape	Upper facial lines/nasolabial folds	Nasolabial folds	Jowls
**2**	Physician	Masseter volume/chin projection and shape	Malar volume loss/upper facial lines	Upper facial lines	Upper eyelid droop
	Patient	Masseter volume	Tear trough	Upper facial lines	Nasolabial folds/upper facial lines
**3**	Physician	Tear trough	Nasolabial folds	Nasolabial folds	Tear trough/upper facial lines
	Patient	Tear trough	Nasal shape	Tear trough/jowls/upper eyelid droop	Upper eyelid droop

Based on results of a pre-meeting survey of the Asian Facial Aesthetics Expert Consensus Group (*N* = 24 responses; the responses of one Australian expert who treated predominantly Caucasian patients were excluded). *Survey question* “For this question, your answer should be based on what the women NEED from an esthetic point of view, NOT on what they actually REQUEST or have treated. In your professional opinion, what are the most critical treatment areas for women aged [18–30/31–40/41–55/>55] years? Choose three and rank them in order.” *Survey question* “Amongst your patients aged [18–30/31–40/41–55/>55] years, what are the three most common presenting esthetic concerns and complaints raised by them (without your or your staff’s guidance or intervention)? Choose three and rank them in order.”

The survey results showed that younger patients (aged ≤40 years) most commonly request the esthetic treatments that improve facial shape and three-dimensionality. While these patients believe that they are merely seeking esthetic improvements, physicians recognize that requested treatments result from the underlying facial structural features common to Asians (described in Table 2) that can contribute to a negative esthetic impact. As patients age, their treatment needs and preferences evolve to address issues associated with aging. Those aged older than 40 years are more likely to request treatments and procedures that improve volume loss, sagging, and wrinkles.

**Table 2:** Skeletal features and related physical characteristics/appearance of the Asian face

Skeletal features in Asians compared with those of Caucasians [8–10, 44–49]	Related physical appearance/clinical features in Asians	Other characteristic facial features of Asians
Increased bitemporal width	Wide forehead	
Increased bizygomatic width	Wide midface	
Increased bigonial width	Wide lower face	
Retruded forehead	Flat forehead, slanted backward	
Retruded orbital rims/shallow orbit	Puffy, heavy eyelids	Epicanthal folds
Low nasal bridge deficient anterior nasal spine	Flat, short nose; appearance of wider intercanthal distance retruded columella	
Medial maxilla retrusion	Under-eye “dark shadow,” concave central midface, perialar recession and nasolabial fold, or shadows on the base of nose, broad nasal width	
Retrusion of pyriform margin	Perioral protrusion	Full upper and lower lips
Bimaxillary protrusion hypoplastic mandible	Retruded chin	

Includes characteristics of ethnic Koreans and Chinese

### Consensus Statements Regarding Evolving Treatment Trends

- The available treatment options, as well as awareness of new treatments and procedures, have increased over the past decade.
- The proportion of younger patients in Asia who present with esthetic concerns has increased over the past 5 years.
- The most common treatment concerns among younger patients (excluding skin concerns) are the result of underlying structural features that can contribute to a negative esthetic impact or relative weakness.

### Facial Anatomy and Morphology in Asians

**Fig. 2:**
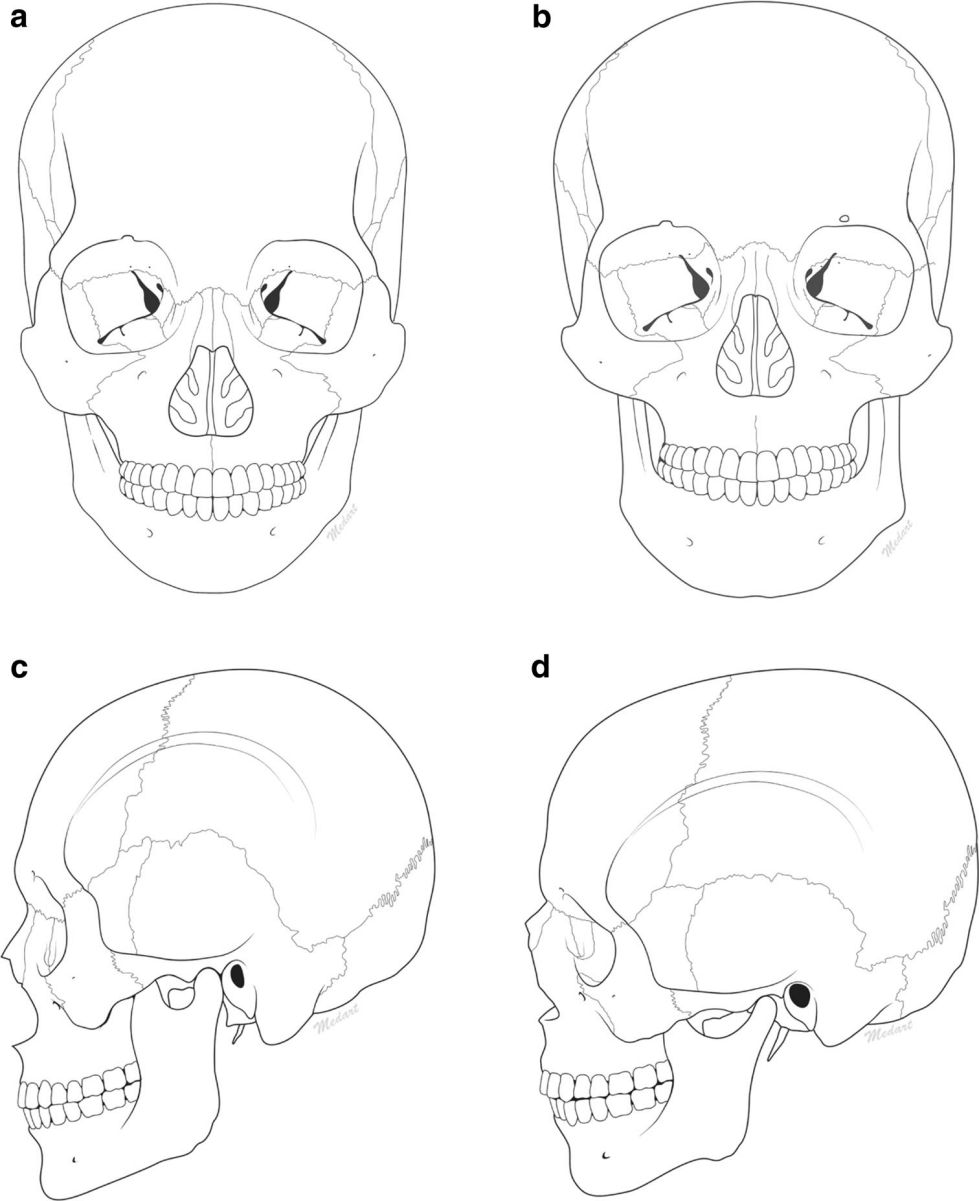
Comparison of Asian (**a**, **c**) and Caucasian (**b**, **d**) skulls. **a**, **b** Anterior view. The Asian skull (**a**) is wider overall, with greater bitemporal, bizygomatic, and bigonial width of the temple, zygoma, and mandible, respectively, compared with those of the Caucasian skull (**b**). **c**, **d** Lateral view. The Asian skull (**c**) has less anterior projection, with a more retruded frontal bone and supraorbital ridge, recessed nasion, infraorbital rim, medial maxilla, maxillary process of the zygoma, anterior nasal spine, and pogonion of the mandible compared with the Caucasian skull (**d**). (Illustrations courtesy of Prof Kim)

**Fig. 3:**
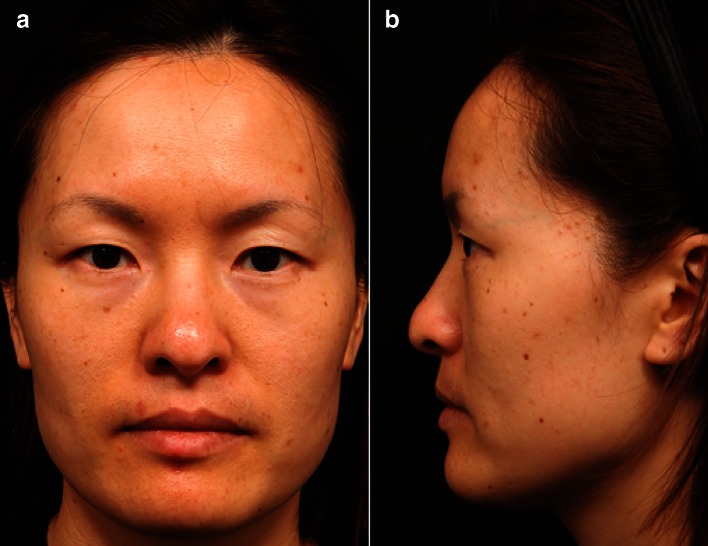
**a** Frontal and **b** lateral views of an Asian female face illustrating some of the morphological features (wide bitemporal, bizygomatic, and bigonial distances; retruded and concave medial maxilla; puffy upper eyelids; epicanthal folds) listed in Table 2. (Photos courtesy of Dr. Steven Liew)

In Asia, patients seek treatment at a relatively young age (Table 1) to address the perceived undesirable facial features that correlate with the underlying characteristic structural anatomical features detailed in Table 2. A thorough knowledge of the patient’s facial anatomy and age-related processes is required to inform facial esthetic treatment. The physical features of the Asian face are related to specific skeletal and morphological features that differ from those of Caucasians (Fig. 2; Table 2). Although there is great diversity due to the ethnic variations that exist among Asian populations, typical facial features can still be identified (Table 2; Fig. 3). Asians tend to have a wide and short face. In profile, the face typically appears flat or, in some cases, even concave. Compared with the Caucasian face, the Asian face is characterized by greater intercanthal width, epicanthal folds, smaller eye fissure length, hooding of the upper eyelid/lateral brow (creating a “puffy” eyelid appearance), smaller oral width, greater mandibular width and a square lower face, and retruded chin. The nose has a flat dorsum, wider base, and less tip projection [9–12, 23, 24]. The lips of Asians tend to be fuller than those of Caucasians, with the upper lip often being more prominent.

Ethnic Asians have a thicker soft tissue layer next to the most lateral point of the ala nasi, compared with Caucasians [25–27]. These factors, together with retrusion of the pyriform margin of the bony structure in Asians, correspond with the hidden columella, wider alar base, and flatter nose characteristically observed in Asians.

### Aging in Asians

#### Skin Aging Processes

Skin aging differs between Asians and Caucasians in several aspects. Pigmentary problems such as lentigines and seborrheic keratosis are particularly common among Asians, but wrinkles tend to manifest 1–2 decades later in Asians than in age-matched Caucasians [13, 14, 28]. A comparison of skin aging in Chinese and French populations living under similar climate conditions indicated that in the Chinese population, wrinkle development followed a biphasic trend, with a slow increase until the ages of 40–50 years, followed by a rapid increase thereafter [13]. By the age of 60 years, the wrinkle intensity in both French and Chinese populations appeared similar. This is likely due to the increased melanin in Asian skin affording a sun protection factor (SPF) of approximately 7, compared with a SPF of 3.4 in Caucasians [29–31].

In another study that compared facial wrinkles among Japanese, Chinese, and Thai women, the wrinkle intensity was greatest among Thais, followed by Chinese, and then Japanese women [32]. With Thailand being the most tropical country of the three and having a higher ultraviolet light exposure, photoaging was the most obvious reason for these findings, but other factors, including language and facial expression, also contributed to differences in wrinkle score between Chinese and Japanese women. Factors other than SPF differences that may also contribute to the reduced skin aging observed between Asians and age-matched Caucasians include skin structure and thickness [31, 33], diets high in antioxidants (e.g., green tea and omega-3 and -6 fatty acids) [34], and smoking rates [35]. Sociocultural aspects, such as skincare practices and muscle use, during language articulation and facial expressions [36] may also contribute to the differential development of dynamic wrinkles between Asians and Western populations.

#### Non-skin Aging/Physiological Aging Processes

In Asians, the physiological facial aging process involves the same dynamic and complex three-dimensional interplay between the overlying soft tissue and its underlying skeletal structures as in Western populations. Facial fat and soft tissue volume loss, deflation and descent, and bony remodeling all give rise to the same common signs of aging [37–40]. Retaining ligaments also play a role: as the ligamentous system attenuates, facial fat descends [41].

Despite the similarities in the physiological processes and characteristics of facial aging among all races, differences in skeletal structural support and in the propensities of facial soft tissue to sag result in slower rates of facial aging in Asians than in Caucasians [14, 32, 42]. In most Asian patients, the dense fat and fibrous connection between the superficial muscular aponeurotic system and the deep fascia reduce midfacial sagging for longer, and the combination of increased superficial fat and thickened dermis lessen the incidence of superficial rhytides [42]. Eventually, however, due to the loss of dermal support (despite the initially thicker skin), the heavier malar fat pad and weaker skeletal support in the Asian face contribute to tissue descent that manifests as facial sagging with aging. Nevertheless, overall, the Asian face retains its youthful appearance for longer due to delayed signs of skin aging and sagging, compared with age-matched Caucasians [14, 15, 32, 42].

### Consensus Statement on Aging

- Asians between the third and sixth decades age well compared with age-matched Caucasians.

## Conclusions

Even within the population described as “Asian” in this manuscript, significant facial morphological differences are seen, which may underlie the scarcity of published treatment recommendations targeted at “Asians” as a group.

Asians have facial anatomical features that may contribute to what they perceive as an esthetically undesirable appearance. Increasing numbers of younger patients in Asia are seeking cosmetic treatment specifically to address these structural issues, as evidenced by the types of treatment that are in highest demand by different age groups [43]. Treatments to achieve esthetic changes in Asians are not aimed at the Westernization, but rather the optimization and beautification of their ethnic features, via the correction of underlying structural characteristics that can contribute to a negative esthetic impact.

In Asians, facial aging manifests differently compared with Caucasians in terms of the time course and observed changes, and Asians who present for anti-aging treatments require different strategies and management techniques from Caucasian patients. The intrinsic sun protection afforded by the pigment in Asian skin delays photoaging, which appears to be an important factor contributing to the perception that Asians age well. Although Asians age better extrinsically, in particular those who present for first treatment at a later age require treatment to address not only the underlying structural features that can contribute to a negative esthetic impact but also cumulative age-related changes. These patients will often require combination treatment and a treatment planning strategy that recognizes the complexity of the interplay between the underlying anatomical state and skin aging.

## Appendix

The Asian Facial Aesthetics Expert Consensus Group also includes Drs. Danru Wang, China; Yan Wu, China; Rashmi Shetty, India; Vandana Chatrath, India; Chytra Anand, India; Adri Prasetyo, Indonesia; Akiko Imaizumi, Japan; Nobutaka Furuyama, Japan; Hideaki Sato, Japan; Hong-Ki Lee, South Korea; Jonathan Nevin Yu, Philippines; Marisa Pongprutthipan, Thailand; and Nantapat Supapannachart, Thailand.
